# Orthodontic screening and treatment timing in preschoolers

**DOI:** 10.1002/cre2.161

**Published:** 2019-02-10

**Authors:** Cristina Grippaudo, Ester Giulia Paolantonio, Valeria Luzzi, Alice Manai, Giuseppe La Torre, Antonella Polimeni

**Affiliations:** ^1^ School of Orthodontics Dental Institute, Catholic University of Sacred Heart, Fondazione Policlinico Universitario A. Gemelli IRCCS Rome Italy; ^2^ School of Orthodontics Dental Institute, Fondazione Policlinico Universitario A. Gemelli IRCCS Rome Italy; ^3^ Department of Oral Science and Maxillofacial Surgery “Sapienza” University of Rome Rome Italy; ^4^ School of Orthodontics Dental Institute, Catholic University of Sacred Heart Rome Italy; ^5^ Department of Public Health and Infectious Diseases “Sapienza” University of Rome Rome Italy; ^6^ Head and Neck Department Policlinico Umberto I University Hospital, Faculty of Medicine and Dentistry, “Sapienza” University of Rome Rome Italy

**Keywords:** malocclusion, orthodontic screening, prevention of malocclusion, treatment timing

## Abstract

Dental and stomatologic problems in childhood need to be diagnosed and managed with multidisciplinary protocols focusing around the children an appropriate prevention, diagnosis, and care program. Therefore, it is paramount to avail of screening tools that provide an indication of in‐depth multidisciplinary diagnostic flow. The aim of this study is to detect and evaluate malocclusion problems and predisposing factors in an Italian preschooler population. Design‐calibrated operators detected data through examination of 1,405 children (706 males and 699 females) aged between 2 and 7 years, in one hospital in Rome and in kindergartens of several Italian cities. Data were collected following Risk of Malocclusion Assessment index criteria. Pearson's chi‐square test (with continuity correction) and Fisher's exact test were the statistical tests conducted (*P* < 0.05). Grade 2 (49.6%) and 4 (21.7%), followed by grade 1 (17.1%), grade 3 (9.3%), and finally grade 5 (2.3%) are the most represented degrees. 53.6% of the cases have a high risk, whereas 32.2% has a low risk and 14.9% has a moderate risk. The risk‐degree correlation is statistically significant (*P* < 0.005). Flawed habits and oral breathing are present in more than a quarter of children. Findings of the study highlighted that early multidisciplinary approach, as well as orthodontic visits and screening in childhood, is necessary to promote normal growth and development of the face and the elimination of potential interferences that may harm these processes.

## 1 INTRODUCTION

According to the National Guidelines for the Promotion of Oral Health and the Prevention of Oral Pathology, published in 2008 by the Italian Ministry of Health and revised in 2013 (Ministero della Salute, 2018a), the incidence of oral pathologies in pediatric age, despite the improvements obtained from preventative and screening campaigns, is still high in Italy.

The promotion of oral health in childhood is part of a broader health prevention and health project that involves many medical specialties, and it is a result of an intentional alliance between the child's parents, sensitized by the need for periodic visits and inspections, and medical specialists to safeguard the overall patient's health. The promotion of specific screening campaigns and guidelines are useful to make significant changes to the overall population trend, with the aim of improving long‐term public health and reducing medical expenses, including a benefit on the country's global economy and health care costs. Pediatric age, in this perspective, becomes the preferred field of action to obtain economic and biological cost/benefit positive ratio of therapy, continuity of care, and creation of a culture of prevention that ensures the population well‐being.

Pediatric orthodontic treatments require a comprehensive and multidisciplinary approach that integrate different disciplines (pediatrics, pedodontics, orthodontics, otolaryngology, allergology, logopedics) to early diagnose and correct craniofacial growth alterations and malocclusion, both apparent and with the potential, already in preschool age. The orthodontist is often the first to diagnose pathologies requiring a multidisciplinary approach, such as obstructive sleep apnea syndrome (Ministero della Salute, 2018b). The Italian Ministry of Health's clinical recommendations state that odontostomatologic problems in childhood need to be identified, diagnosed, and managed with multidisciplinary protocols that are designed to put the child into an appropriate prevention, diagnosis, and care program.

In this perspective, it is important to use screening tools that provide, when needed, an indication of in‐depth multidisciplinary diagnostic flow.

The Baby Risk of Malocclusion Assessment (ROMA) index (Table 1) is an orthodontic treatment needs index for orthodontic screening in children aged between 2 and 6 years, in complete deciduous or early mixed dentition. It is replicable, has internal validity, and provides a degree of severity and need for treatment proportional to the severity of the detected condition. It is complete and takes into consideration systemic, skeletal, dental, and functional problems (Grippaudo, Paolantonio, Pantanali, Antonini, & Deli, 2014).

**Table 1:** The Baby Risk of Malocclusion Assessment index

**Baby ROMA index**		
		Grade
*Systemic problems*		
	Maxillofacial trauma with condylar fracture	5a
	Maxillofacial trauma without condylar fracture	2a
	Congenital syndromes/malformations	5b
	Postural/orthopedic problems	2c
	Medical or auxological conditions	2d
	Inheritance of malocclusion	2e
*Craniofacial problems*		
	Facial or mandibular asymmetries	4f
	TMJ dysfunctions	4g
	Outcomes of trauma or surgery on the craniofacial district	5j
	Maxillary hypoplasia or mandibular hyperplasia (OVJ < 0 mm)	4 k
	Maxillary hypoplasia or mandibular hyperplasia (OVJ > 0 mm)	2 k
	Maxillary hyperplasia or mandibular hypoplasia (OVJ > 6 mm)	3 h
	Maxillary hyperplasia or mandibular hypoplasia (3 mm < OVJ < 6 mm)	2 h
*Dental problems*		
	Caries and early loss of deciduous teeth	4 l
	Scissor bite	4 m
	Crossbite >2 mm or lateral shift	4n
	Crossbite <2 mm or no lateral shift	2n
	Displacement >2 mm	3o
	Displacement >1 mm—absence of diastema	2o
	Open bite >4 mm	3p
	Open bite >2 mm	2p
	Hypodontia/hyperdontia more than two teeth	4q
	Hypodontia/hyperdontia less than two teeth	3q
	Overbite >5 mm	2r
	Poor oral hygiene	2t
*Functional problems*		
	Parafunction	2v
	Thumb/finger sucking habits	2w
	Oral breathing/OSAS	2x
None of the problems listed above (*N*)		1

*Note*. OSAS: obstructive sleep apnea syndrome; ROMA: Risk of Malocclusion Assessment; TMJ: temporomandibular joint.

In this research, we integrated the Baby ROMA index by two tables, which are organized similar to those for prevention of caries and oral pathology in growing children (Ministero della Salute, 2018a). These preventive orthodontic tables, simple for compilation and reading, help to detect the degree of risk and to provide treatment indications. They are also useful for communicative use with pediatricians and parents (Tables 2 and 3). The tables identify a risk scale (low, moderate, high) that corresponds to the need of orthodontic treatment and multidisciplinary assessment, behavioral, therapeutic, and preventive advice required to avoid a worsening of the detected malocclusion. In fact, the table for recommended interventions also indicates the need for ear, nose, and throat (ENT) and logopedic diagnostics.

**Table 2:** Risk assessment

Factors	Low risk	Moderate risk	High risk
*Functional*			
Suction (pacifier, thumb, lower lip)			Yes
Respiratory problems (oral breathing, adenoid and/or tonsillar hypertrophy, otitis, roncopathy)			Yes
OSAS			Yes
Facial asymmetry			Yes
Functional limitation of opening or deviation			Yes
*Occlusal*			
Deep bite		Yes	
Open bite			Yes
Lateral or anterior crossbite			Yes
Maxillary protrusion		Yes	
Mandibular Protrusion			Yes
No diastema		Yes	
Displacement		Yes	
*Dental*			
Cavities			Yes
Teeth missing			Yes
Supernumerary teeth			Yes
Dental trauma		Yes	
*Protective*			
Normal eruption	Yes		
Normal function	Yes		
Normal growth	Yes		

*Note*. OSAS: obstructive sleep apnea syndrome

**Table 3:** Recommended interventions

Risk	Periodical control	Preventive interventions	Therapeutic interventions
Low	Every 12 months	Oral Hygiene, proper nutrition, no sucking habits	Periodical surveillance, ENT evaluation for breathing problems
Moderate	Every 6 months	Oral Hygiene, proper nutrition, no sucking habits, treatment for dental trauma	Periodical surveillance, ENT evaluation for breathing problems
High	Every 3 months	Oral Hygiene, proper nutrition, no sucking habits, treatment for dental trauma	Periodical surveillance, ENT evaluation for breathing problems, orthodontic treatment, myofunctional therapy

*Note*. ENT: ear, nose, and throat.

The aim of this research is to conduct an epidemiological study on the presence of malocclusion problems and predisposing factors in an Italian preschooler population.

## 2 MATERIALS AND METHODS

### 2.1 Index description

The Baby ROMA index (Grippaudo et al., 2014) is an index for the early assessment of the risk of malocclusion. It was created and validated in 2014 starting from ROMA index (Grippaudo, Paolantonio, Deli, & La Torre, 2007) so that it could be used specifically for children in complete deciduous dentition or premature mixed dentition because current used indices did not include this age group. Only Summers' (1972) Occlusal Index provided different scores for deciduous, mixed, and permanent dentition, but its drawback is not taking into account skeletal and dental problems. The Baby ROMA index was introduced to analyze not only dental factors but also skeletal, functional, and systemic factors affecting malocclusion in preschooler children, providing support to the clinician in the diagnostic phase and in the choice of the therapeutic timing.

### 2.2 Study design and sample recruitment

**Figure 1:**
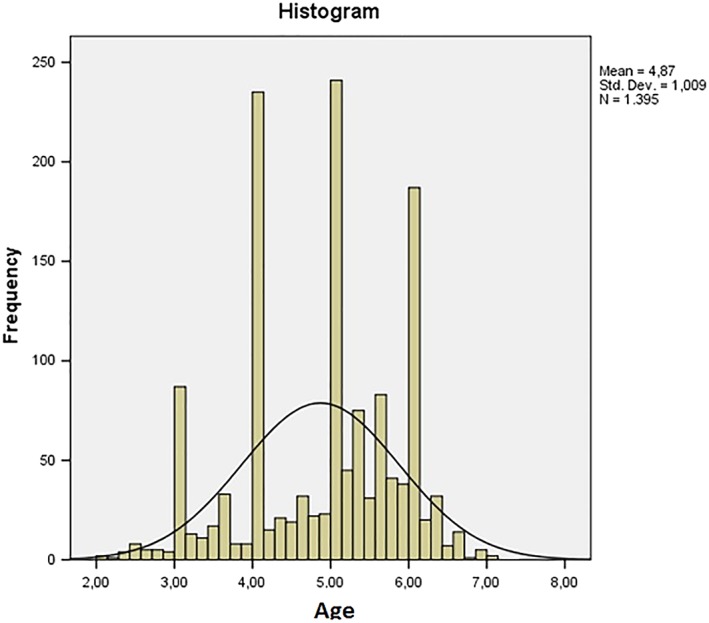
Histogram of age distribution

A transversal observational study was performed applying the Baby ROMA index and the preventive orthodontic tables. Data were collected by visiting 1,405 children between the ages of 2 and 7, including 699 females and 706 males (Figure 1). Patients were examined at the Dental Clinic of Fondazione Policlinico A. Gemelli IRCSS in Rome, at pediatric clinics and kindergartens. All children observed presented a complete deciduous dentition or an early mixed dentition (permanent first molars, one or two permanent central in the upper and/or lower jaw). Noncooperating children and children with ongoing orthodontic treatment were excluded from the study.

### 2.3 Clinical observation and ethical concerns

Artificial illumination, a gauge (to measure overjet and overbite), and a tongue depressor were the instruments used for the inspective examination. In the execution of the visit, the interview with parents was a priority, both in the anamnesis and in the communication of the results of the screening performed. Young children were involved to complete the visit, but more detailed information was provided by parents regarding date of birth, relevant medical and auxological conditions, syndromes and congenital abnormalities, postural and orthopedic abnormalities, inheritance of malocclusion, previous significant traumas to the facial and dental district, flawed habits and nonnutritive suction, bruxism and grinding, poor hygiene habits, previous treatments of carious lesions, and previous dental assessments. In the observations made at pediatric clinics, the pediatrician contributed to the collection of general medical anamnestic information.

Permission was granted from the head teacher in kindergartens and from pediatricians, after they viewed a presentation letter of the project. All parents and/or tutors of children agreed for the study protocol. Children's parents from school signed a statement of informed consent prior to the clinical observation. The study has been performed according to the Declaration of Helsinki, without harm, coercion, nor exploitation for participants.

### 2.4 Reproducibility criteria and calibration

The children's visit and data collection were performed by different calibrated operators. First of all, all the examiners attended a course of ROMA and Baby ROMA index, given by two instructors (EGP and CG). The course was completed with clinical training. Intraexaminer and interexaminer reproducibility were calculated to verify the reliability of the index using *K* test. The intraexaminer reproducibility was tested comparing the data of 20 children examined by the same operator. The interexaminers were tested with the same group of 20 children collected by another operator. At the *K* test, a high correlation between operators was observed; therefore, the index is highly reproducible. The *K* values of intraexaminer correlation ranged between 0.643 and 1.00, and the *K* values of interexaminers correlation were between 0.773 and 1.00.

### 2.5 Data collection

The Baby ROMA Index and the preventive orthodontic tables were used for data collection. This index saves all the signs of malocclusion and the presence of risk factors.The collection of the different items in each sample subject allowed to perform an accurate and precise epidemiological investigation, so as to define not only the risk of malocclusion but also the skeletal, occlusal and functional characteristics most represented in the population being examined. Furthermore, the dental formula of each individual in the sample was collected. It has been integrated by inserting information regarding any dental anomalies present, specifying the type of anomaly (agenesis, supernumerary, fusion, gemination) and the site (element number).

In the data collection form of the Baby ROMA Index, the degree of risk is indicated by the item with a higher numerical value, but all the items observed in the subject were reported. Children's data were stored in a database according age and gender.

In particular, regarding the baby ROMA index, it was assessed as follows:

- Prevalence of the grade in the sample,
- Prevalence of the risk in the sample,
- Most frequent malocclusion in the sample,
- Distribution of the items of grade 2 in the sample (%),
- Distribution of the items of grade 3 in the sample (%),
- Distribution of the items of grade 4 in the sample (%),
- Risk‐degree correlation.

The aim of this evaluation consists in investigating the early signs of malocclusion from a complete deciduous dentition phase to an early mixed‐dentition phase. All the risk factors were taken into account to forecast the possibility of developing a dental or skeletal problem during growth, even if clinical signs of malocclusion are not so evident yet.

### 2.6 Data analysis

The statistical analysis was performed by the Epidemiology Center of the Department of Public Health and Infectious Diseases, Faculty of Medicine, and Surgery, La Sapienza University, Rome, Italy. The significance was verified by Pearson's chi‐square test, with continuity correction and Fisher's exact test for nonnumeric variables (Tables 4 and 5). The level of significance was set at 5% (*P* < 0.05).

**Table 4:** Risk * grade cross tabulation

			Grade			
			1	2	3	4
Risk	Low risk	Count	237	199	1	4
		Within risk (%)	53.7	45.1	0.2	0.9
	Moderate risk	Count	1	177	29	2
		Within risk (%)	0.5	84.3	13.8	1.0
	Hish risk	Count	1	316	100	297
		Within risk (%)	0.1	42.5	13.5	40.0
*Total*		*Count*	*239*	*692*	*130*	*303*
		*Within risk (%)*	*17.1*	*49.6*	*9.3*	*21.7*

**Table 5:** Chi‐square tests

	Value	*df*	Asymptotic significance (2 sided)
Pearson chi square	887.629	8	.000
Likelihood ratio	1020.588	8	.000
Linear‐by‐linear association	602.443	1	.000

## 3 RESULTS

**Figure 2:**
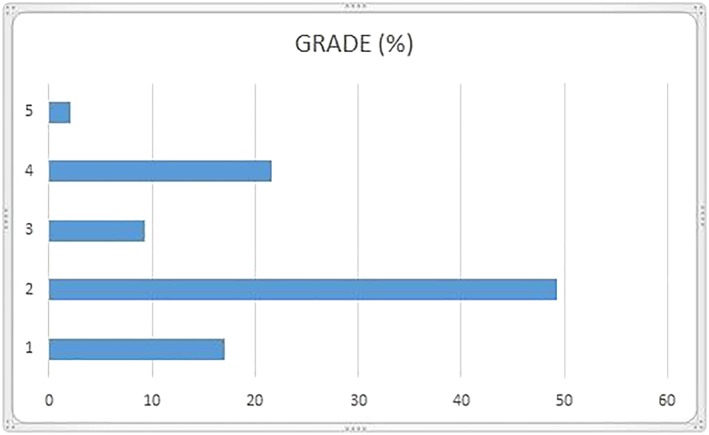
Graphic of risk grade

**Figure 3:**
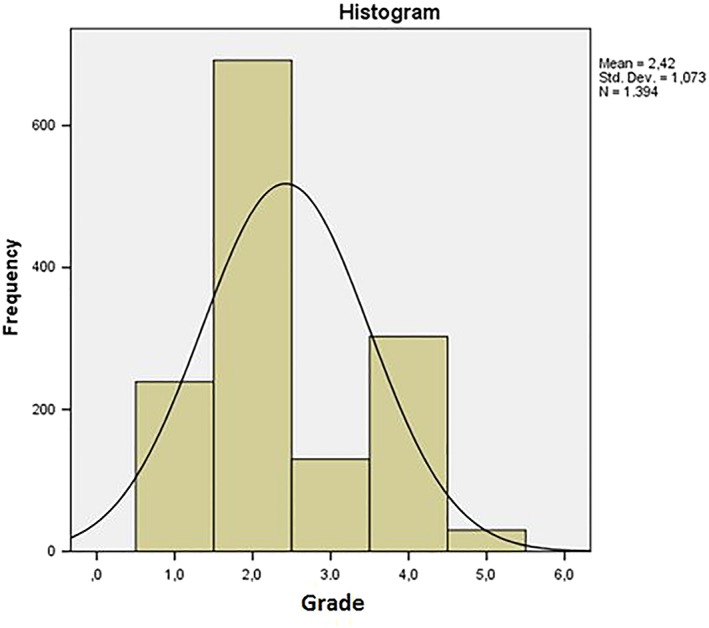
Histogram of orthodontic risk distribution

The analysis of the data, as in Figures 2 and 3, shows that the most represented degrees are 2 (49.6%) and 4 (21.7%), followed by grade 1 (17.1%), grade 3 (9.3%), and finally grade 5 (2.3%).

**Figure 4:**
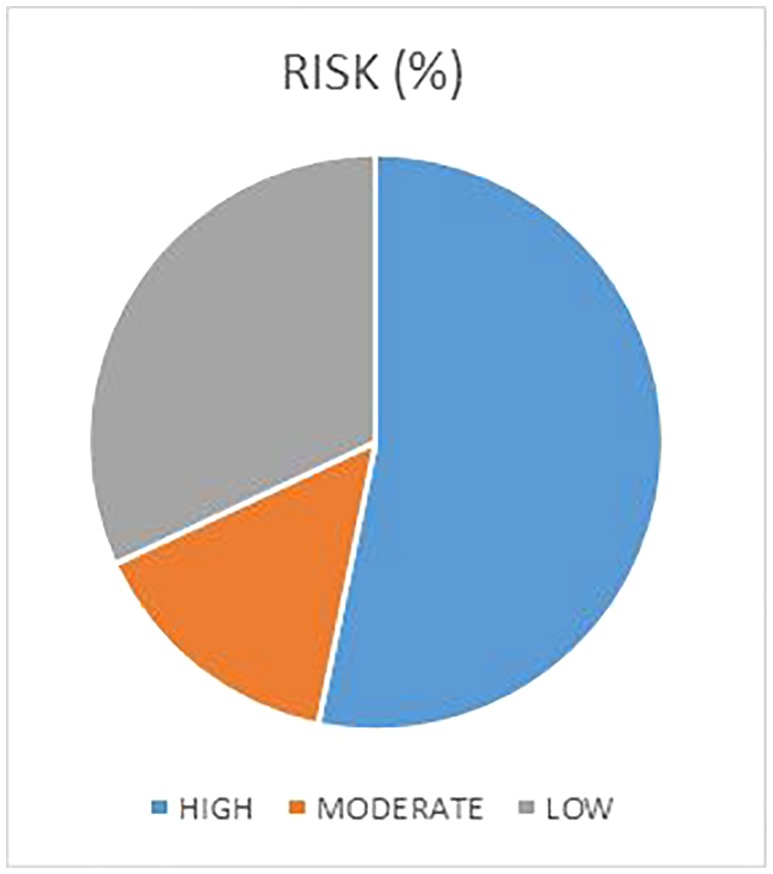
High, moderate and low risk distribution

**Figure 5:**
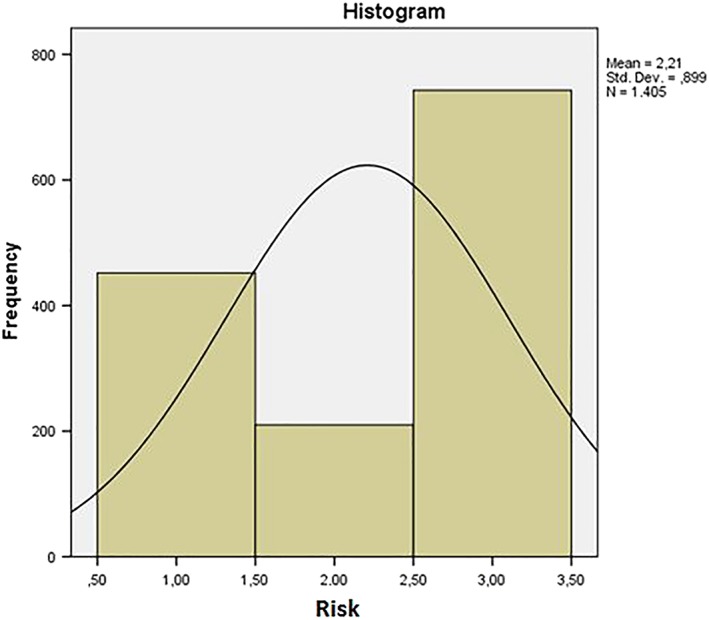
Histogram of orthodontic risk distribution (for high, moderate, and low risk)

**Figure 6:**
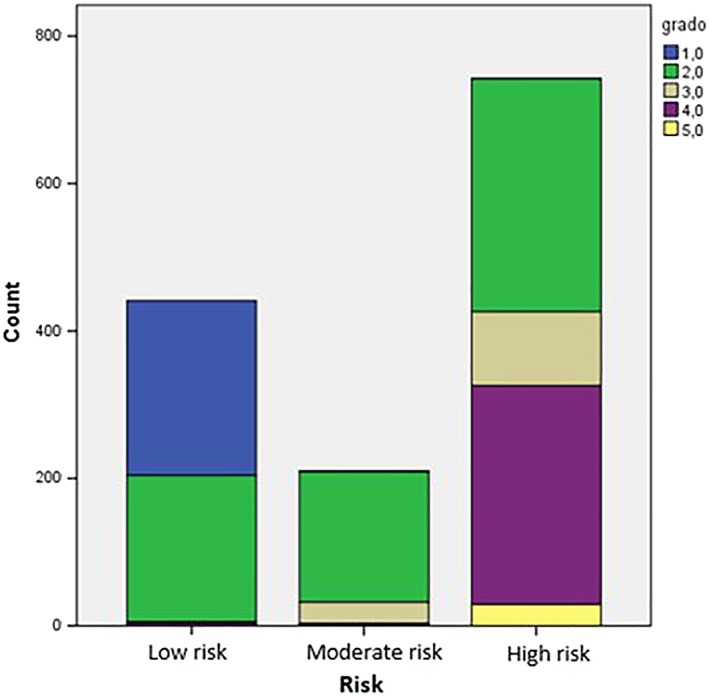
Grades distribution

The high risk is present in 53.6% of the cases (Figures 4, 5, 6); about one third of the children visited had a low risk (32.2%), and the remaining children had a moderate risk (14.9%). The risk‐degree correlation is statistically significant (*P* < 0.005).

**Figure 7:**
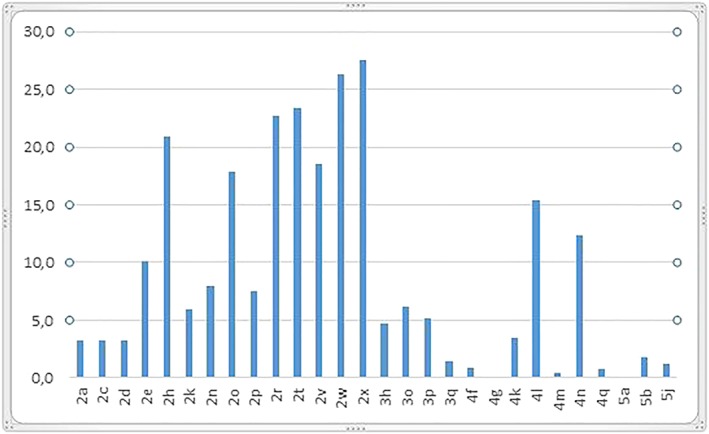
Histogram of grades frequency

More than a quarter of children had bad habits (27.5%), oral breathing (26.3%), and increased overjet (25.6%) of which 20.9% between 3 and 6 mm and 4.7% greater than 6 mm. About a quarter of the sample had increased overbite (22.7%). Poor oral hygiene was widespread, with a prevalence of 23.4%, as well as decay of deciduous teeth with 15.4%. 12.3% of the cases having a crossbite with a mandibular shift (Figure 7). There was also a significant prevalence relating to the absence of diastemas (17.9% in grade 2) and crowding (6.2% in grade 3), open bite greater than 2 mm (5.5%) and greater than 4 mm (7.2%).

**Figure 8:**
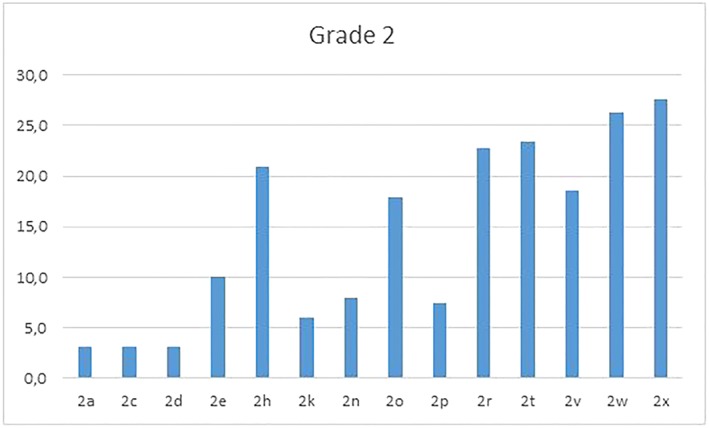
Histogram of functional problems distribution

**Figure 9:**
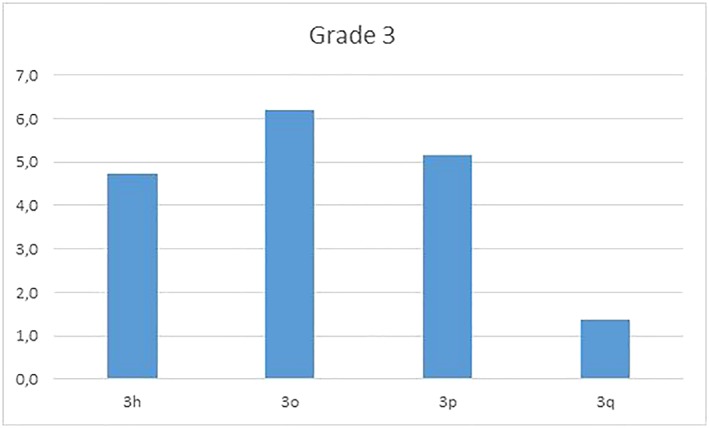
Graphic of grade 3 problems

**Figure 10:**
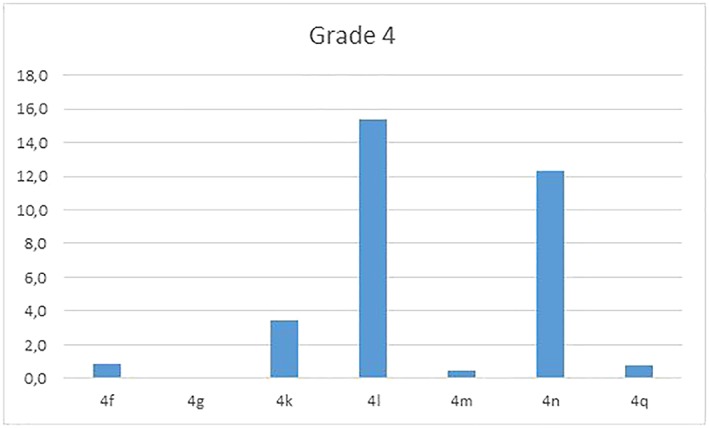
Graphic of grade 4 problems

Figures 8, 9, 10 reflect distribution of the items of grades 2, 3, and 4, respectively.

## 4 DISCUSSION

Prevention is the first strategy of contemporary medicine that must be applied to reduce the prevalence of diseases and reduce the biological costs and expense of health treatments (Emerich & Wojtaszek‐Slominska, 2010). Even in orthodontics, it would be desirable to apply this rule as early interception of bad habits, oral breathing therapy, the interceptive orthodontic treatment of precocious malocclusions that have recently appeared and are simple to correct, could be of great benefit to preschool patients (Bell, Dean, McDonald, & Avery, 2011; Kurol, 2002).

Therefore, our work is oriented on the evaluation of the preschoolers' treatment need. Knowing the prevalence of the malocclusions found and their correct timing, we can establish the proper treatment path for this patient target.

Most of the occlusal problems found in our work were grade 2 (49.6%), which includes parafunctions, bad habits, oral breathing, and low‐grade malocclusions that sometimes require closer monitoring and even timely speech therapy or ENT treatment, due to their higher risk of worsening in severe malocclusion if untreated. In fact, pacifier sucking, baby bottle sucking, and especially thumb sucking frequently causes protrusion of the upper incisors and the premaxilla, atypical swallowing (Larsson, 1994; Larsson, 2001), anterior open bite, and posterior crossbite (Castelo, Gavião, Pereira, & Bonjardim, 2010; Melink, Vagner, Hocevar‐Boltezar, & Ovsenik, 2010; Warren, Bishara, Steinbock, Yonezu, & Nowak, 2001). The posterior crossbite is due to a low positioning of the tongue due to sucking, as the lack of tongue thrusting on the palate and the increased activity of the muscles of the cheeks cause an alteration of muscle pressure on the upper arch (Carrascoza, Possobon R de, Tomita, & de Moraes, 2006; Ovsenik, 2009).

The influence of breathing on the craniofacial morphology, such as the obstruction of the upper airways resulting in mouth breathing, changes the pattern of craniofacial growth (Harvold, Tomer, Vargervik, & Chierici, 1981) with facial features and dentition typical: long face, contraction of the upper dental arch, high‐arched palate, gummy smile, dental malocclusion both Class 2 and Class 3 (Harari, Redlich, Miri, Hamud, & Gross, 2010). In mouth breathing, it was observed that, compared with the general population, there was a higher prevalence of posterior crossbite, of anterior open bite and Class 2 malocclusion (Souki et al., 2009).

It is instead better to treat, if there is compliance, the cases of grade 4 (prevalence 21.7%) with high degrees of risk, in which the malocclusion is already manifest, whereas in the cases of grade 3 (9.3%), without functional alterations, there is only a need for close monitoring, followed by revaluation. In the case of grade 1, periodic checks must be scheduled.

The study shows that over 50% of the children who visited had a high degree of risk or already had a malocclusion that could worsen over time (grade 4). Additionally, some had signs or alterations of orofacial and respiratory functions closely related to the development of malocclusions (grade 2) and which, if not removed or resolved, may result in malocclusion.

The association of functional alterations, breathing problems, and malocclusions often requires multidisciplinary intervention and the cooperation of several medical specialists. The early intervention of the specialist (ENT, allergist, pediatric dentist, orthodontist, speech therapist) with simple and targeted therapies is necessary to rebalance the stomatognathic system and to ensure that it grows harmoniously. An important role is played by the pediatrician in intercepting and diagnosing the malocclusion at an early age. In fact, the malocclusion is a gradual process that is established already in the first years of life and stabilizes or worsens in the following years if not treated (Luzzi et al., 2017; Grippaudo, Paolantonio, Deli, & La Torre, 2008; Grippaudo, Pantanali, Paolantonio, Saulle, Latorre, & Deli, 2013 Sep; Grippaudo, Pantanali, Paolantonio, Grecolini, Saulle, La Torre, Deli, 2013 Dec). The main dental, occlusal, and functional problems must be identified with awareness and must be intercepted by the pediatrician who sends the patient to the orthodontist or to the pediatric dentist as a formal policy of referring (Badri, Saltaji, Flores‐Mir, & Amin, 2014; Luzzi et al., 2017). Doing so would prevent the development of skeletal and dental malocclusion or hinder worsening malocclusions already established—an event that would then require longer and more complex treatments to be solved.

## 5 CONCLUSION

In light of the findings of this research, we believe in the necessity of early orthodontic visits and screening in childhood, pediatrician training, and counseling with families because, in our opinion, these are effective interventions to prevent and early diagnose not only malocclusions but also breathing problems and speech disorders. Measures to prevent malocclusion should be based on all the multidisciplinary interventions useful to promote normal growth and development of the face and eliminate potential interferences that may harm these processes. The collaboration among pediatrician, orthodontist, pediatric dentist, and other therapists is essential for the oral health care in children and for creating a system of reference to specialized figures. Thus, we hope to create a formal protocol aiming to guide patients from the pediatrician to the orthodontist or to the pediatric dentist, in order to include the children in a specialized diagnostic and therapeutic path.

## 6 BULLET POINTS

Why this paper is important to pediatric dentistry:

1. Dental and occlusal problems in childhood need to be diagnosed and managed with multidisciplinary protocols.
2. Precocious diagnosis and intervention are pivotal aspects of prevention.
3. The different risk of malocclusion grades diffusion highlighted by our findings shall encourage a comprehensive medical approach aiming to foster normal growth and development of the stomatognathic system.

## CONFLICT OF INTERESTS

The authors declare they have no conflict of interest.
